# Human helminth therapy to treat inflammatory disorders- where do we stand?

**DOI:** 10.1186/s12865-015-0074-3

**Published:** 2015-03-26

**Authors:** Helena Helmby

**Affiliations:** Department of Immunology and Infection, Faculty of infectious and Tropical Diseases, London School of Hygiene and Tropical Medicine, Keppel street, London, WC1E 7HT UK

**Keywords:** Helminths, Inflammation, Regulation, Autoimmunity

## Abstract

Parasitic helminths have evolved together with the mammalian immune system over many millennia and as such they have become remarkably efficient modulators in order to promote their own survival. Their ability to alter and/or suppress immune responses could be beneficial to the host by helping control excessive inflammatory responses and animal models and pre-clinical trials have all suggested a beneficial effect of helminth infections on inflammatory bowel conditions, MS, asthma and atopy. Thus, helminth therapy has been suggested as a possible treatment method for autoimmune and other inflammatory disorders in humans.

## Introduction

### The hygiene hypothesis

One of the first reports suggesting a link between environmental living conditions and allergic disease originates already from the 1970s [1] where members of a predominant urban white community was reported to have higher levels of allergic disease, compared to a rural indigenous community where levels of viral, bacterial and helminth infections were much higher. A similar observation was made by Strachan a decade later, reporting that children with elder siblings were less likely to develop hay fever leading to the original Hygiene hypothesis proposal, hypothesising that reduced exposure to infections in early childhood owing to a combination of diminishing family size, improved living standards and higher levels of personal hygiene might result in increased risk of developing allergic disease later in life [2]. The hypothesis has since been extended to include other types of immune-mediated and inflammatory disorders such as autoimmune diseases (e.g. multiple sclerosis (MS)) and inflammatory bowel disease (IBD) [3,4], all conditions with a sharp increase in prevalence throughout westernised high-income countries in the last few decades [5]. This increase in immune-mediated disorders correlate with urbanisation and economic development but which specific aspects of the westernised lifestyle that are responsible have not yet been clearly defined. Changes in air pollution levels, increased indoor exposure to allergens and general improvement of living standards have all been implicated. In addition, childhood exposure to changes in intestinal microbiota [6] and a variety of pathogenic microbes, including helminths, have also been suggested to play a part. Helminth infections, in particular intestinal worms, were up to a few decades ago common in all parts of the world but have now been more or less eradicated in high-income countries, despite still being a major public health problem in the rest of the world. The ability of worms to modulate the host response to a state of what can be described as “anti-inflammatory tolerance” together with the sharp increase in inflammatory disorders, paralleled with the decrease in helminth infections in high income countries, has generated a strong interest in the possibility that worms, or their products, could be used as new anti-inflammatory treatments.

### Helminth-induced immune responses

Work in mouse models have clearly established that immunity to intestinal helminths is dependent on a Type 2 cytokine response, involving the secretion of IL-4, IL-5, IL-9 and IL-13 and the subsequent activation of intestinal mast cells, eosinophils, goblet cells, enterocyte proliferation and intestinal contractility (reviewed in [7]). In addition, Type 2 responses promote the “walling off ” of eggs or larvae in tissues via granuloma formation as well as promoting tissue repair mechanisms, an important component of infections with large metazoan parasites as evidenced by the fact that a failure to mount a Type 2 response is generally associated with increased pathology and tissue destruction [8]. In addition to a Type 2 response, various immunoregulatory mechanisms are induced, including increases in regulatory T cell (T reg) numbers and IL-10 and/or TGF-β levels, resulting in a highly anti-inflammatory environment. Studies have highlighted the importance of IL-10 in controlling pathology associated with helminth infections as IL-10 deficient mice suffer higher mortality and/or morbidity [9,10], whilst depletion of T regulatory cells *in vivo* result in increased immune responses and parasite clearance [11,12]. Overall there is substantial evidence that the increased regulatory activity during helminth infection need to strike a fine balance between protection against pathology and clearance of the infection. Since helminths are so good at generating immunoregulatory mechanisms, the question naturally arises as to whether helminth replacement therapy may have a therapeutic role to play in the treatment of autoimmune and allergic disorders.

## Review

### Helminth therapy in humans

To date two species of helminths have been tested for human helminth therapy as a clinical treatment, *Trichuris suis*, the pig whipworm, and the human hookworm *Necator americanus*. After ingestion of *T.suis* ova (TSO), the eggs hatch and the worms colonise the caecum and colon of the human gut for only a short period of time (weeks) meaning that treatments need to be repeated at intervals, however, this species-specificity and lack of chronic infection is beneficial in the sense that it also removes any wider public health issues. Larvae of the human hookworm *Necator*, however, are administered percutaneously and migrate through the vasculature and lungs to the small intestine where they survive by feeding on blood from the mucosa, giving rise to long lasting infections (years) and may at higher doses cause clinical symptoms such as gastrointestinal symptoms and anemia. In its natural state this infection is a major public health problem across the globe and large-scale deworming programs are in place to combat the morbidity associated with natural infection [13].

### Helminth therapy used to alleviate intestinal inflammation

Several studies in animal models have demonstrate that intestinal helminth infections are able to inhibit the development of intestinal inflammation (reviewed in [14,15]) and the first clinical studies of helminth therapy in humans started some 10–15 years ago with the use of the pig whipworm *Trichuris suis*. In initial safety studies patients with Ulcerative colitis (UC) or Crohn’s disease were given viable, embryonated *T.suis* eggs (TSO) and not only was the treatment well tolerated but a significant disease remission was observed and although the beneficial effect was temporary, repeated doses of TSO sustained this clinical improvement suggesting a promising new therapy for IBD [16,17]. A placebo–controlled, double blind, randomised trial in Ulcerative colitis patents followed, showing significantly improved disease activity index in TSO treated patients compared to placebo, although the remission rate was no different between the two groups [18]. Further development and safety testing of TSO under GMP was performed and a small randomized double-blind placebo controlled study reported that Crohn’s patients receiving a single dose of up to 7500 TSO did not show any short (2 weeks) or long term (6 months) adverse effects [19] opening up the field towards larger clinical trials. To date at least six clinical trials using TSO in Crohn’s or UC patients have been registered as recruiting, ongoing or completed. However, in October 2013 Coronado biosciences announced in a press release that the results from the first larger study (TRUST-1, trial identifier NCT01576471), a Phase 2 clinical trial evaluating TSO in 250 US patients with moderate-to-severe Crohn’s disease, did not meet its primary endpoint of improving responses, either in terms of improving disease activity index or remission rates, although a non-significant improvement was noted in patients with a more severe disease score [20]. Shortly after, a second Corona press release announced the discontinuation of the Phase 2 study of 240 European Crohn’s patients (FALK, trial identifier NCT01279577) after an independent monitoring committee recommended its discontinuation due to “lack of efficacy” [21]. No further data has been released from either study. Although the clinical trial results for TSO therapy in Crohn’s patients are disappointing, results from several Ulcerative colitis trials are still eagerly awaited.

A second approach to helminth therapy has been the slightly more controversial use of the human hookworm *Necator*, a pathogen responsible for much of the morbidity associated with intestinal helminth infections around the globe. In a small trial where 9 Crohn’s patients were infected with 25–50 larvae and followed over 20 weeks, 7 patients experienced improved disease score while 2 experienced a worsening effect [22]. A second study examined hookworm versus placebo therapy in a cohort of 20 coeliac disease (gluten allergy) patents followed by wheat challenge after 20 weeks. The dose of 5–10 larvae was generally well tolerated and immunological analysis demonstrated reduced inflammatory cytokine (IFN-γ and IL-17) responses in duodenal biopsies from hookworm compared to placebo-treated patients [23], however there was no difference in the symptomatic response to wheat challenge with all subjects experiencing the same levels of clinical symptoms regardless of treatment [24]. Further clinical trails of using hookworm infections in coeliac patients are still expected.

### Helminth therapy and allergy

Another field of much interest in recent years is whether helminth therapy may be useful in reducing allergic symptoms. Several studies from helminth endemic areas have suggested that certain helminth infections may protect against allergy and asthma but a systematic review of 33 published studies concluded that there was no overall protective effect of helminth infections in general on asthma [25]. However, concurrent hookworm infection was associated with a protective effect, which was infection-intensity dependent. In contrast, concurrent *Ascaris lumbricoides* infection, another common intestinal nematode infection, was associated with a significantly increased risk of asthma. This is particularly interesting given the fact that both hookworms and *Ascaris* pass through the lungs during their migration to the intestine but only *Ascaris* is being known as causing tropical pulmonary eosinophilia syndrome, due to its high allergenicity [25], thus demonstrating that only certain specific helminth species are likely to be beneficial from a helminth therapy perspective.

Studies on the relationship between helminth infection and atopy have also generated mixed results with both positive and negative associations depending on the species of worms involved [26] and deworming studies in helminth endemic communities have either shown no evidence for increased skin prick test (SPT) reactivity [27], or increased SPT reactivity [28,29]. However, allergen SPT reactivity may also be influenced by worm infections due to the fact that many helminth antigens crossreact with common allergens and it may be that the release of helminth antigens from dying worms after anti-helminthic treatment may increase reactivity temporarily. In this context it is important to recognize that several highly immunogenic helminth proteins share structural relationships with a number of common allergens, for example, IgE cross-reactivity has been demonstrated between helminth (e.g. filarial and Ascaris) tropomyosins and the tropomyosins of house dust mite (Der p 10) and cockroaches (Bla g 7) suggesting that helminth infections may well be able to enhance allergic reactivity. The number of potentially cross-reactive proteins shared among helminths and allergens has been suggested to be very extensive, with 40% of 499 molecularly defined allergen families having homologs in helminth parasite genomes [30-32].

In the light of a large body of literature suggesting some protective benefits of helminth infections on allergy and asthma a few human helminth therapy trials in asthma/allergy have been published. The first one, a randomized trial using 8 doses of TSO, or placebo, at an interval of 21 days in 100 patients with grasspollen-induced allergic rhinitis showed no significant effect on rhinitis symptoms, grass-specific IgE levels, or SPT reactivity, despite inducing *T.suis*-specific antibody responses and gastrointestinal symptoms [33]. Similarly, a small randomized safety study in individuals with allergic rhinoconjunctivitis treated with hookworm larvae or placebo, and followed for 12 weeks, reported no significant effects on lung function, SPT or rhinconjunctivitis symptoms, despite clear evidence of hookworm-induced responses such as increased eosinophilia and gastrointestinal symptoms [34]. Another small randomized control trial in patients with asthma again showed no significant benefit of hookworm infection on clinical symptoms, bronchial responsiveness or SPT reactivity [35]. It should be noted, however, that both the hookworm studies were small studies with 15 and 16 patients in each group, respectively, and using low doses of larvae (10), while the timing of infection in relation to pollen season may also need to be adjusted to reach optimal affects. As such, further trials are required in order to draw any firm conclusions on the potential benefits in using helminth therapy against allergies and asthma.

There are a number of potential reasons why the results from human trials have not generated more positive data. A large number of animal studies have demonstrated a potent ability of a variety of helminth infections to reduce allergic reactivity in mice and rats (reviewed in [14]), however the vast majority of studies have shown this as an ability to prevent the development of allergic reactivity after exposure to helminths, and only a handful have reported the ability for the infections to impact an already established allergic reactivity. Furthermore, a few animal studies have also reported the inability of helminth infections to alter such an established allergic response. As such, most of the experimental data available suggest that once the allergic reaction is established helminth infections can do little to alter this, raising the inevitable question whether there is any true benefit to gain from helminth therapy in already allergic individuals. Regardless, in terms of the disappointing clinical trials in humans there are still question remaining surrounding whether optimal timing of treatment, the dose and whether systemic versus non-systemic infections may play an important part. TSO is entirely restricted to the intestine and may not induce sufficient systemic response to alter the environment in the lungs or other parts of the body. The human hookworm *Necator* however does migrate through the lungs at the early stages of infection but here the question remains if the dose (10 larvae) is sufficient to induce enough of a response. Needless to say virtually all animal studies have used significantly higher infection doses than may be viewed as safe to ever use in humans. Finally, the timing of infection versus the onset of seasonal allergy may need to be investigated as the immunomodulatory effect of helminth infection may take longer time to develop than what was measured in the trials to date. In addition, the use of low dose trickle infections may also improve immunmodulatory activity over time and warrants further investigation.

### Other uses for helminth therapy

Animal studies using the MS mouse model of experimental autoimmune encephalomyelitis (EAE) has suggested a protective effect of helminth infections on CNS disease progression [36,37] and a prospective study demonstrated that 12 MS patients infected with a variety of helminth infections had significantly fewer relapses and lower MRI activity when compared to 12 non-helminth infected MS patients over a time period of 4.5 years [38]. In a follow up study it was further shown than when these patients received anti-helminthic treatment their clinical presentation deteriorated and this was associated with a reduction in IL-10 and TGF-β, and an increase in IFN-γ and IL-12 secretion from MBP peptide stimulated PBMCs [39] thus providing further support that helminth therapy may be of some benefit in MS patients. Subsequently, a phase 1 study for TSO treatment in 5 multiple sclerosis patients reported fewer new lesions during and up to two months after TSO treatment as well as increased serum levels of IL-4 and IL-10 [40]. Several Phase 1/2 clinical trials using TSO or hookworm in MS patients are currently registered as recruiting or ongoing (NCT00645749, NCT01413243, NCT01470521). In addition to MS a number of clinical trials are currently registered for the use of TSO in patients with psoriasis, autism and rheumatoid arthritis.

### Potential for helminth products as new drugs

Helminths secrete a rich mixture of proteins, carbohydrates and lipids, collectively named excretory-secretory (ES) products, into their surrounding environment and many of these ES products have been found to exhibit a variety of immunomodulatory activities. The best characterized product to date is the ES-62 molecule from the filarial nematode *Acanthocheilonema vitae* (reviewed in [41]), a glycoprotein with potent ability to skew dendritic cells towards promoting Th2 and inhibiting Th1 and Th17 polarisation. In addition, ES-62 is able to inhibit mast cell activation and induce IL-10 secretion from B cells and macrophages. ES products from a variety of other helminths have also been shown to drive Th2 differentiation and induce *de novo* differentiation of T regulatory cells, suggesting a therapeutic potential for inflammatory disorders. Indeed, animal studies have demonstrated that a variety of ES products can protect against allergen-induced airway hypersensitivity in mice, limiting peri-bronchial inflammation by inhibiting eosinophil and neutrophil infiltration of the lungs while increasing T regulatory cell numbers and IL-10 secretion. Moreover, animal studies have shown the potent ability of various ES products to inhibit intestinal inflammation in colitis models, the development of Th1-dependent type 1 autoimmune diabetes in NOD mice, reducing the development of EAE in the mouse model of MS and blocking the induction of collagen-induced arthritis (reviewed in [41-43]). Taken together, all this evidence suggests an exciting potential for new drug discoveries to be made. However, much work remains before such products can be taken to the clinic, as most of the ES products remain to be characterized in detail and any problems with potential antigenicity and/or allergenicity needs to be resolved, such as the development of non-immunogenic mimetics [41].

## Conclusions

Without doubt there is overwhelming evidence from animal studies that helminth infections exert strong immunomodulatory activity and are able to inhibit, alter and modify other ongoing immune responses. In addition, human crossectional studies have established that many chronic helminth infections in endemic communities are associated with the induction of regulatory and anti-inflammatory networks which may act to inhibit inflammatory responses such as autoimmune and allergic reactions. However, to translate this into clinical helminth therapy forms have proved less successful in the few published clinical trials conducted so far. It may be that for worms to be successful in controlling inflammation we need to be exposed to them before the onset of the inflammatory condition or even that we need to be exposed to them at a young age to allow our immune system to co-develop together with them. In recent years substantial interest has been generated in the field of inflammation and autoimmunity regarding the impact of the composition of the intestinal microbiota and its role in shaping our immune responses both in early life and later [44], including the importance of diet in maintaining a healthy gut community [45] but it remains to be established whether worms form a vital part of this “healthy intestinal community”. Regardless, some promising data has been achieved using human helminth therapy but many questions remains to be investigated, such as the appropriateness of the species of helminths used, whether infections should be systemic or localized, whether the dose should be light or heavy, of acute or chronic duration, and the role of host genetics. In addition, the use of helminth-derived anti-inflammatory molecules is yet to be tested on a clinical scale but may be offering a less controversial, and perhaps more palatable, promising new avenue of anti-inflammatory drug development.
